# NEDD4L-induced β-catenin ubiquitination suppresses the formation and progression of interstitial pulmonary fibrosis *via* inhibiting the CTHRC1/HIF-1α axis

**DOI:** 10.7150/ijbs.57247

**Published:** 2021-07-25

**Authors:** Lin Chen, Yang Yang, Haiying Yan, Xiaying Peng, Jun Zou

**Affiliations:** Department of Respiratory and Critical Care Medicine, Sichuan Provincial People's Hospital, University of Electronic Science and Technology of China, Chengdu 611731, P.R. China

**Keywords:** Interstitial pulmonary fibrosis, Lung fibroblasts, NEDD4L, β-catenin, Ubiquitination, Collagen triple helix repeat containing protein 1, Hypoxia-inducible factor-1α

## Abstract

Interstitial pulmonary fibrosis (IPF) is a severe progressive lung disease with limited therapeutic options and poor prognosis. Initially, we found the downregulated level of neural precursor cell expressed developmentally down-regulated 4-like protein (NEDD4L) in IPF-related expression microarray dataset, and this study was thus performed to explore the molecular mechanism of NEDD4L in IPF. The expression of NEDD4L was subsequently validated in lung tissues of IPF patients and mouse models. Then, mouse primary lung fibroblasts (LFs) were collected for *in vitro* functional experiments, with CCK-8, Transwell, and immunofluorescence assays used to examine the viability, migration, and differentiation of LFs. The *in vitro* findings were further assessed using *in vivo* mouse models. The expression of NEDD4L was down-regulated in lung tissues of IPF patients and mouse models. Overexpression of NEDD4L restricted the formation and progression of IPF in mice and attenuated the proliferative, invasive and differentiative abilities of LFs. Further, NEDD4L halted LFs activity by enhancing β-catenin ubiquitination and down-regulating the CTHRC1/HIF-1α axis. Also, *in vivo* experiments then validated that NEDD4L silencing repressed β-catenin ubiquitination and activated the CTHRC1/HIF-1α axis, thereby aggravating IPF in mice. NEDD4L may suppress the formation and progression of IPF through augmenting β-catenin ubiquitination and inhibiting the CTHRC1/HIF-1α axis.

## Introduction

Interstitial pulmonary fibrosis (IPF) represents a common type of IPF and is characterized by deposition of the extracellular matrix and aberrant fibrotic remodeling of the lung, leading to progressive loss of lung function and ultimately death 1. Although it has been considered rare, the prevalence of IPF is similar to that of stomach and testicular cancers 2. IPF occurs more commonly in the elder and the median age of patients at diagnosis is about 65 years 3. From a pathophysiological view, IPF is currently considered as a consequence of multiple interacting genetic and environmental risk factors, closely associated with repetitive local micro-injuries to ageing alveolar epithelium 1. In spite of the complicated and somewhat unpredictable disease course, the median survival time of IPF from diagnosis is 2 to 4 years 4.

Further, myofibroblasts have been recognized as the main cell type able to stimulate the accumulation of extracellular matrix in fibrotic diseases, and targeting the proliferation and differentiation of fibroblast has thereby been considered as a promising therapeutic strategy for IPF 5, 6. Neural precursor cell expressed developmentally down-regulated 4-like protein (NEDD4L) is a member of ubiquitin ligases NEDD4 family and serves as a tumor suppressor in various cancers 7. Interestingly, a recent case has suggested that the conditional deletion of NEDD4L in lung epithelial cells could trigger pulmonary fibrosis and bronchiolization, which were key features of IPF 8.

On the other hand, a previous report has pointed out that collagen triple helix repeat containing protein 1 (CTHRC1) could promote the development of pulmonary fibrosis and the level of CTHRC1 was elevated in lung tissues of IPF 9. Of note, our bioinformatics analysis indicated that NEDD4L expression was negatively correlated with CTHRC1 expression in lung tissue of IPF. Hence, CTHRC1 might has a critical role to confer in the regulatory mechanism of NEDD4L in IPF. Furthermore, it has been reported that CTHRC1 could up-regulate the expression of hypoxia-inducible factor-1α (HIF-1α), and HIF-1α has been highlighted for its pulmonary fibrosis-promoting ability 10, 11. In this sense, we speculated that NEDD4L may regulate the progression of pulmonary fibrosis through the CTHRC1/HIF-1α signal axis. Moreover, it has been established that the Wnt/β-catenin signal axis was activated in pulmonary fibrosis, and β-catenin could bind to the CTHRC1 promoter region 12. Taken together, in this study, we proposed a hypothesis that NEDD4L may suppress the formation and progression of IPF through augmenting β-catenin ubiquitination and inhibiting the CTHRC1/HIF-1α axis.

## Methods and Materials

### Ethics statement

The study was conducted in accordance with the Declaration of Helsinki and approved by the Ethics Committee of Sichuan Provincial People's Hospital, University of Electronic Science and Technology of China. Detailed study aims as well as planned procedures were explained to all patients, who were subsequently provided with signed informed consent documentation. Animal experiments were approved by the Animal Care and Use Committee of Sichuan Provincial People's Hospital, University of Electronic Science and Technology of China and performed in accordance with *Guide for the Care and Use of Laboratory Animals* published by the National Institutes of Health.

### Bioinformatics Analysis

Pulmonary fibrosis-related GSE10667 expression microarrays were retrieved from the Gene Expression Omnibus (GEO) database (https://www.ncbi.nlm.nih.gov/gds), which contained 8 samples of lung tissues from IPF patients and 15 of normal lung tissues. The Limma R language package (http://www.bioconductor.org/packages/release/bioc/html/limma.html) was then utilized to identify differentially expressed genes in IPF samples, with “|log fold change (FC)| > 1, *p* value < 0.05” as the screening threshold.

#### Sample collection

Lung tissues with IPF were collected from abnormal area indicated by histological examinations in biopsy of IPF patients (n = 35, 19 men and 16 women with an average age of 44 ± 9 years), and normal lung tissues from normal organ donations (n = 28, 13 men and 15 women with an age range of 46 ± 10 years). All patients met the American Thoracic Society/European Respiratory Society's diagnostic criteria for lung fibrosis. Apart from outpatient and telephone follow-ups, patients were required to have a lung biopsy every month for the first three months after discharge, then every three months, and then every six months two years after the operation. Part of the tissue samples were quickly frozen in liquid nitrogen and stored at -80°C, and the other part was made into paraffin sections for subsequent experiments.

### Immunohistochemistry

After dewaxing, hydration, washing, endogenous peroxidase neutralization and antigen recovery, the 4 μm-thick tissue sections were blocked with goat serum at 37°C for 1 h, and then incubated overnight with anti-β-catenin (ab32572, 1:500, Abcam, Cambridge, UK) and anti-CTHRC1 antibodies (16534-1-AP, 1:20, Proteintech, Chicago, IL, USA) at 4°C. On the second day, the sections were washed with phosphate buffered saline (PBS) and processed with a two-step assay kit (PV-9000, GBI, USA). Subsequently, the sections were stained with DAB kit (PW017, Sangon Biotech, Shanghai, China) and hematoxylin, dried at 65°C, and sealed, followed by observation using an optical microscope (CX23, Olympus, Japan).

### Establishment of an IPF mouse model

A total of 60 C57BL/6 male mice (4-6 weeks old, provided by the Institute of Zoology, Chinese Academy of Sciences, Beijing, China) were housed with a 12-h light/dark cycle at 21 - 23℃. After one week of adaptation, the mice were randomly divided into 2 groups (Sham: n = 6, IPF: n = 54). IPF mice were anesthetized with 5% pentobarbital sodium and given Bleomycin by intratracheal suction (0.005 U/g, dissolved in 50 mL normal saline, APP Pharma-ceuticals, Schaumburg, IL, USA); and sham-operated mice was intratracheally administered the same amount of normal saline as controls. Two weeks later, Resistance and Compliance Plethysmographs (Shanghai Yuyan Instruments Co., Ltd., Shanghai, China) were adopted to evaluate lung functions.

Further, 54 IPF mice were randomly divided into 9 groups and subjected to tail vein injection of different lentiviral (lv) particles: IPF (2 mL saline as a control), IPF + NC (2 mL lv-NC particles), IPF + NEDD4L (2 mL lv-NEDD4L particles), IPF + sh-NC (2 mL lv-sh-NC particles), IPF + sh-NEDD4L (2 mL sh-NEDD4L lentiviral particles), IPF + DMSO (2 mL DMSO), IPF + PR-619 (2 mL deubiquitinase inhibitor PR-619), IPF + PR-619 + sh-NC (2 mL PR-619 and 2 mL lv-sh-NC lentiviral particles), and IPF + PR-619 + sh-NEDD4L (2 mL PR-619 and 2 mL sh-NEDD4L lentiviral particles) groups. The titer of lentiviral particles (Genechem, Shanghai, China) was 1×10^9^ TU/mL, and the tail vein injection was performed on the 5th day of Bleomycin induction and then every 4 days. Three weeks later, the mice were euthanized to collect the lung tissues, which were then incubated with 4% paraformaldehyde (PFA) and embedded in paraffin for subsequent experiments.

### Hematoxylin and eosin (H&E) staining

Lung tissues were fixed with 4% PFA, embedded with paraffin, and sectioned with an automatic microtome (RM2265, Leica, Germany). After deparaffinization and rehydration, the sections were stained following the instructions of H&E staining kit (AR1180, Boster Biological Technology, Chicago, IL, USA), and images were photographed during observation using an optical microscope (Eclipse E200, Nikon, Tokyo, Japan). Then, two pathologists double-blindly observed the morphological changes of the tissue with an optical microscope, and the pathological changes of the tissue was based on the degree of edema, inflammatory cell infiltration, hemorrhage and necrosis, as previously described 13.

### Masson staining

After deparaffinization and rehydration, the sections were stained ​​utilizing the Masson staining kit (G1340, Solarbio Life Sciences, Beijing, China) and observed using an optical microscope (Eclipse E200, Nikon). Images were photographed and analyzed with image J software.

### Hydroxyproline assay

Lung tissues was dried at 100°C for 24 h, hydrolyzed with 0.5 μM acetic acid and 0.1 mg/mL pepsin under hypoxia, and placed with vials of vacuum, followed by overnight incubation at 4°C. Then, the tissued were examined with the hydroxyproline kit (BC0255, Solarbio) to measure the content of hydroxyproline.

### Culture of primary fibroblast

The six to eight-week-old C57BL/6 mice were euthanized. Collected lung tissues were cut into pieces of 1 mm^3^, and treated with digestion buffer containing 30 mg collagenase IV, 1 g fetal bovine serum (FBS), 96 mL Hanks buffer Solution, and 4 mL 0.25% trypsin (Sigma, St. Louis, MO, USA). Then, 1 h later, the tissue suspension was filtered through a cell strainer, and centrifuged to collect the precipitation, which was resuspended in DMEM (Sigma) and centrifuged again to collect the supernatant. After centrifugation, the obtained cells were identified as lung fibroblasts (LFs) utilizing Vimentin immunohistochemical staining and resuspended with DMEM supplemented with 10% FBS, 100 U/mL penicillin and 100 μg/mL streptomycin. The cell resuspension was then incubated in a medium containing TGF-β1 (10 ng/ml, Sigma) at 37℃ and 5% CO_2_.

### Cell grouping and transfection

The LFs in logarithmic growth period were digested with 0.25% trypsin, seeded in a 6-well plate at 1×10^5^ cells/well for 24-h culture, and treated with 10 μM MG132 (protease enzyme inhibitor, M7449, Sigma) upon the cell confluence reached 60-75%. Then, transient cell transfection was performed following the instructions of Lipofectamine 2000 (Invitrogen, Carlsbad, CA, USA). Cells were divided into several groups and transfected with plasmids expressing NEDD4L or carrying small interfering RNA (siRNA) targeting NEDD4L or overexpressing CTHRC1 or corresponding NCs, or treated with DMSO or β-catenin inhibitor or deubiquitinase inhibitor PR-619. The transfection plasmids were synthesized by and purchased from Genechem. The medium was renewed 6 h after the transfection, and the cells were collected after 48 h of culture for subsequent experiments.

### Cell Counting Kit-8 (CCK-8) assay

The LFs in the logarithmic growth phase were seeded in a 96-well plate at a density of 5×10^3^ cells/well and incubated with 10 μL of the CCK-8 reagent (C0038, Beyotime Biotechnology, Shanghai, China) at 37°C for 1 h. Then, a Micro-plate Reader (Bio-Tek Instruments, Winooski, VT, USA) was used to detect the absorbance of each well at 450 nm. Cell viability (%) = average absorbance of the treatment group/average absorbance of the control group × 100%.

### Transwell assay

The LFs in the logarithmic growth phase were suspended in serum-free medium at a density of 5×10^4^ cells/cm^2^, and then seeded in the upper chamber of the Biocoat Invasion Chamber (BD Falcon, Franklin Lakes, NJ, USA) covered with Matrigel gel (50 μL/well). The lower chamber was added with 800 μL of medium supplemented with 10% FBS. After 24-h incubation at 37°C and 5% CO_2_. the chamber was washed and stained with 0.1% crystal violet for 30 min, followed by PBS washing to remove non-invaded cells. Then, 10 fields of view were randomly selected for observation and cell counting under an inverted microscope (BX63, Olympus, Tokyo, Japan).

### Immunofluorescence assay

The LFs in the logarithmic growth period were fixed in 4% PFA at room temperature for 30 min, permeabilized, and then blocked, followed by incubation with anti-α-SMA antibody (#19245, 1:200, CST, Framingham, MA, USA) at 4°C overnight. Next, the cells were incubated with Alexa Fluor® 555-coupled goat anti-rabbit antibody (#60839, 1:50, CST) in the dark, and the nucleus was stained with 4′6-diamidino-2-phenylindole (DAPI, Roche Molecular Biochemicals, Basel, Switzerland) for 5 min. Then the cells were observed with a fluorescence microscope (80i, Nikon, Japan) and images were photographed.

### Immunoprecipitation

HEK293 cells (CRL-1573, ATCC, USA) were lysed with protease-inhibitor cock-tail (Roche) supplemented with cell lysis buffer (Sigma). The lysate was centrifuged to collect the supernatant, which was then incubated with Protein A/G Sepharose beads (Santa Cruz Biotechnology, Santa Cruz, CA, USA) for 30 min at 4°C. Meanwhile, protein A/G sepharose beads were incubated for 10 min in PBS containing 0.1% Triton X-100 and protease-inhibitor with the following antibodies: NEDD4L (ab240753, 1:50, Abcam), CTHRC1 (ab264410, 1:50, Abcam), β-catenin (ab32572, 1:50, Abcam), Ubiquitin (ab209263, 1:30, Abcam), and IgG (ab197767, 1:50, Abcam). Then, the treated supernatant was incubated with the antibody-treated beads at 4°C for 3 h to collect the precipitation by centrifugation. After washing with PBS containing 0.1% Triton X-100 and protease-inhibitor, the precipitation was resuspended in SDS buffer for Western blot assay.

### Chromatin immunoprecipitation (ChIP) assay

Based on the instructions of the Ion 550TM ChIP kit (A34537, Ion Torrent, Thermo Fisher Scientific, Waltham, MA, USA), cells of each group were harvested when the cell confluence reached 70-80% and incubated with 10 μM MG132 and 1% formaldehyde, followed by 10-min fixing at room temperature. Then, the cross-linking of internal DNA and protein was broken by ultrasonic treatment into fragments and centrifuged to collect the supernatant. The supernatant was divided and added into three tubes, respectively incubated overnight at 4°C with positive control antibody RNA polymerase II, negative control antibody normal human IgG, and rabbit anti-β-catenin (ab32572, 1:50, Abcam). The mixtures were treated with Protein Agarose/Sepharose to precipitate the endogenous DNA-protein complex, followed by centrifugation to collect the precipitation, i.e. the non-specific complex, which was then de-cross-linked at 65°C overnight. The DNA fragment was obtained by phenol/chloroform extraction and purification, and RNA extraction and quantitative reverse-transcription polymerase chain reaction (qRT-PCR) was performed to measure the expression of β-catenin.

### qRT-PCR

Total RNA was extracted by Trizol reagent (15596026, Invitrogen) from tissues and cells. Subsequently, RNA was reverse-transcribed into cDNA utilizing the A3500 Reverse Transcription System (Promega, Madison, WI, USA), and the cDNA was amplified utilizing the Fast SYBR Green PCR Master Mix (4385612, Life technologies, Carlsbad, CA, USA). The primers were listed in Supplementary Table 1. Then, the ABI 7900 Fast Real-Time PCR system (ABI, Foster City, CA, USA) were used for qRT-PCR determination. Further, relative quantification method was used to calculate the relative transcription level (normalized to GAPDH) of target genes: _△△_Ct = _△_Ct experimental group - _△_Ct control group, _△_Ct = Ct (target gene) - Ct (internal control).

### Western blot assay

Total protein was extracted from cells and tissues utilizing RIPA lysis buffer (MT0066, Beijing Biolab Technology Co., Ltd., Beijing, China), followed by the determination of total protein concentration using BCA detection kit (ab152036, Abcam). Then, 30 μg cell lysate was mixed with corresponding 5× loading buffer (Beyotime Institute of Biotechnology, China), denatured at 100°C for 10 min. The protein was then separated by 10% sodium dodecyl sulfate polyacrylamide gel electrophoresis (SDS-PAGE), electro-transferred to polyvinylidene fluoride (PVDF) membrane, and blocked with 5% skimmed milk powder in Tris buffered saline (TBS). Subsequently, the membrane was incubated overnight at 4°C with diluted rabbit primary antibodies, including anti-NEDD4L (ab240753, 1:2000, Abcam), anti-β-catenin (ab68183, 1:500, Abcam), anti-CTHRC1 (16534-1-AP, 1:500, Proteintech), anti-HIF-1α (20960-1-AP, 1:1000, Proteintech), anti-collagen 1 (14695-1-AP, 1:1000, Proteintech), anti-Fibronectin (#26836, 1:1000, Proteintech) and anti-β-actin (#8457, 1:1000, CST). After washing, the membrane was further incubated for 1 h with horseradish peroxidase (HRP)-labeled IgG secondary antibody (#7074, 1: 2000, CST, USA). The enhanced chemiluminescence detection kit was then utilized to visualize the protein bands, and the gray level of protein bands was quantified with the Image J 1.48 software (National Institutes of Health, Bethesda, MD, USA).

### Statistical analysis

Data in this study were processed utilizing GraphPad Prism 8.0 (GraphPad Software, La Jolla, CA, USA). Measurement data were summarized as mean ± standard deviation. Independent sample *t* test was applied for comparison between data of two groups; one-way analysis of variance (ANOVA) with Tukey's post-hoc test was performed for comparison among data of multiple groups. Pearson analysis was applied for the correlation between indicators. Moreover, the level of significance was *p* < 0.05.

## Results

### NEDD4L is downregulated in lung tissue of IPF and represses the development of IPF

**Figure 1:**
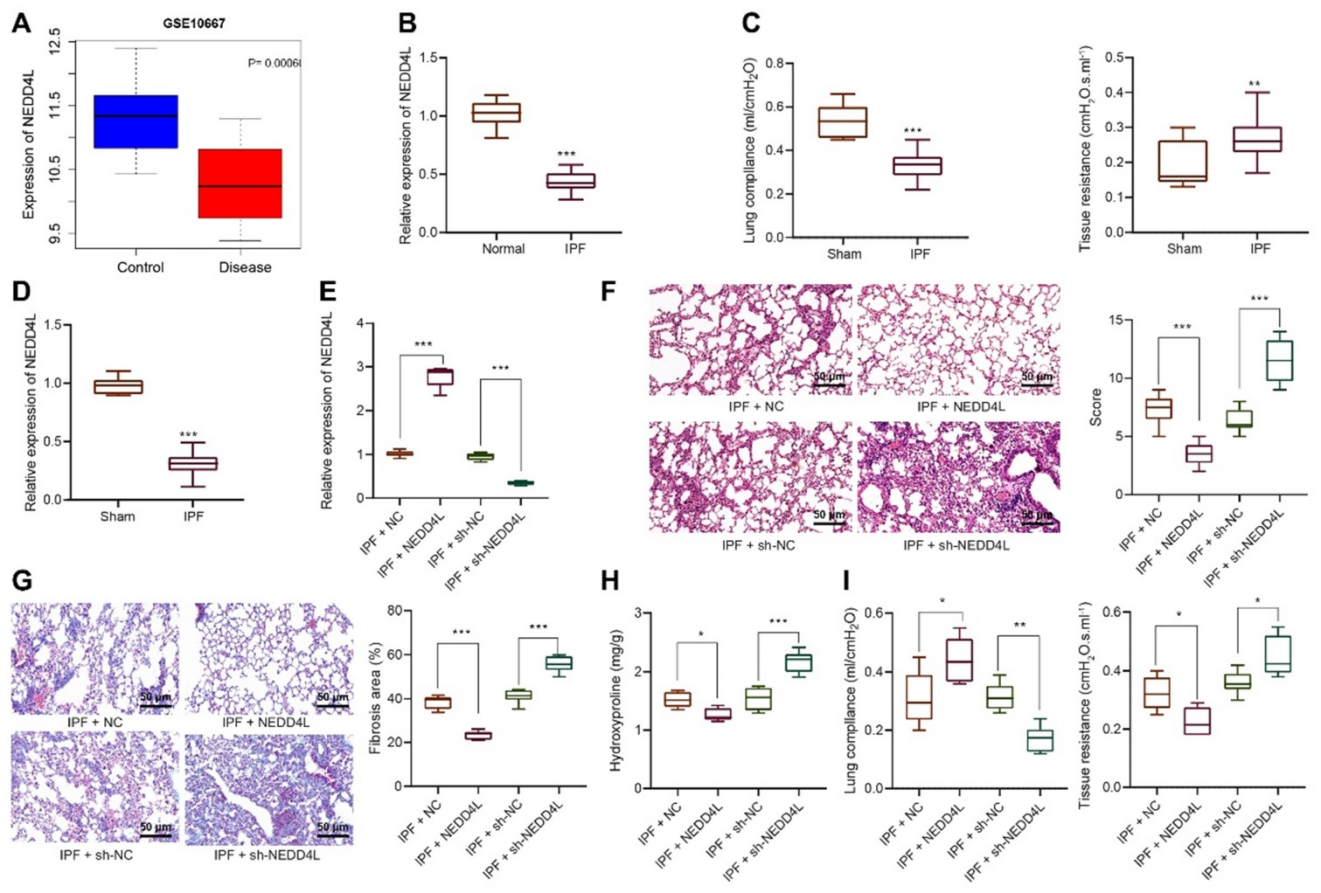
** The expression and effect of NEDD4L in lung tissues of IPF patients and mouse models.** A: Analysis of NEDD4L expression in IPF and normal tissues in GSE10667 microarrays; B: qRT-PCR to determine the NEDD4L expression in lung tissues of IPF and normal samples (35 cases of IPF and 28 cases of normal lung tissues); C: Resistance and compliance plethysmograph to evaluate changes in lung function in IPF mice (sham group: n=6, IPF group: n=54); D: qRT-PCR to determine the expression of NEDD4L in the lung tissue of IPF mice; E: qRT-PCR to measure the expression of NEDD4L in the lung tissue of IPF mice after NEDD4L overexpression/silencing; F: Representative images and scores of the pathological changes of the lung tissue of IPF mice of each group (n = 6); G: Masson staining to determine the degree of lung tissue fibrosis in IPF mice; H: Hydroxyproline content in the lung tissue of IPF mice; I: Resistance and compliance plethysmograph to detect the lung function of IPF mice. * *p* <0.05, ** *p* < 0.01, *** *p* < 0.001 versus the Normal/Sham/IPF + NC group. Measurement data were summarized as mean ± standard deviation. Independent sample *t* test was applied for comparison between data of two groups; one-way ANOVA was adopted for comparison among data of multiple groups. The experiment was performed in triplicates.

Through bioinformatics analysis we identified the down-regulated expression of NEDD4L in lung tissue of IPF (Figure 1A). Results of qRT-PCR further indicated such down-regulation as compared with normal lung tissues (Figure 1B). Thus, we speculated that NEDD4L might regulate the progression of pulmonary fibrosis. Through the resistance and compliance plethysmograph assay, we found attenuated lung compliance as well as augmented lung resistance (Figure 1C), which indicated that our establishment of IPF mouse model was successful. Further, results of qRT-PCR revealed that the expression of NEDD4L in the lung tissue of the IPF mice was down-regulated (Figure 1D). Moreover, NEDD4L overexpression and shRNA plasmids could respectively lead to elevated and decreased level of NEDD4L in the lung tissues of IPF mice (Figure 1E). Through H&E and Masson staining, we found that NEDD4L overexpression could repress inflammatory cell infiltration, alveolar wall thickening, collagen fiber deposition, and lung tissue lesion; whereas NEDD4L knockdown could exacerbate these symptoms (Figure 1F, G). Further, NEDD4L overexpression led to reduced content and silencing NEDD4L led to increased content of hydroxyproline in the mouse lung tissues (Figure 1H). Moreover, the overexpression of NEDD4L protected lung functions in IPF mice, while silencing NEDD4L led to the opposite (Figure 1I). Taken together, these results indicated that NEDD4L overexpression could repress the progression of IPF in mouse model.

### NEDD4L overexpression attenuates the proliferative, invasive and differentiative abilities of LFs

**Figure 2:**
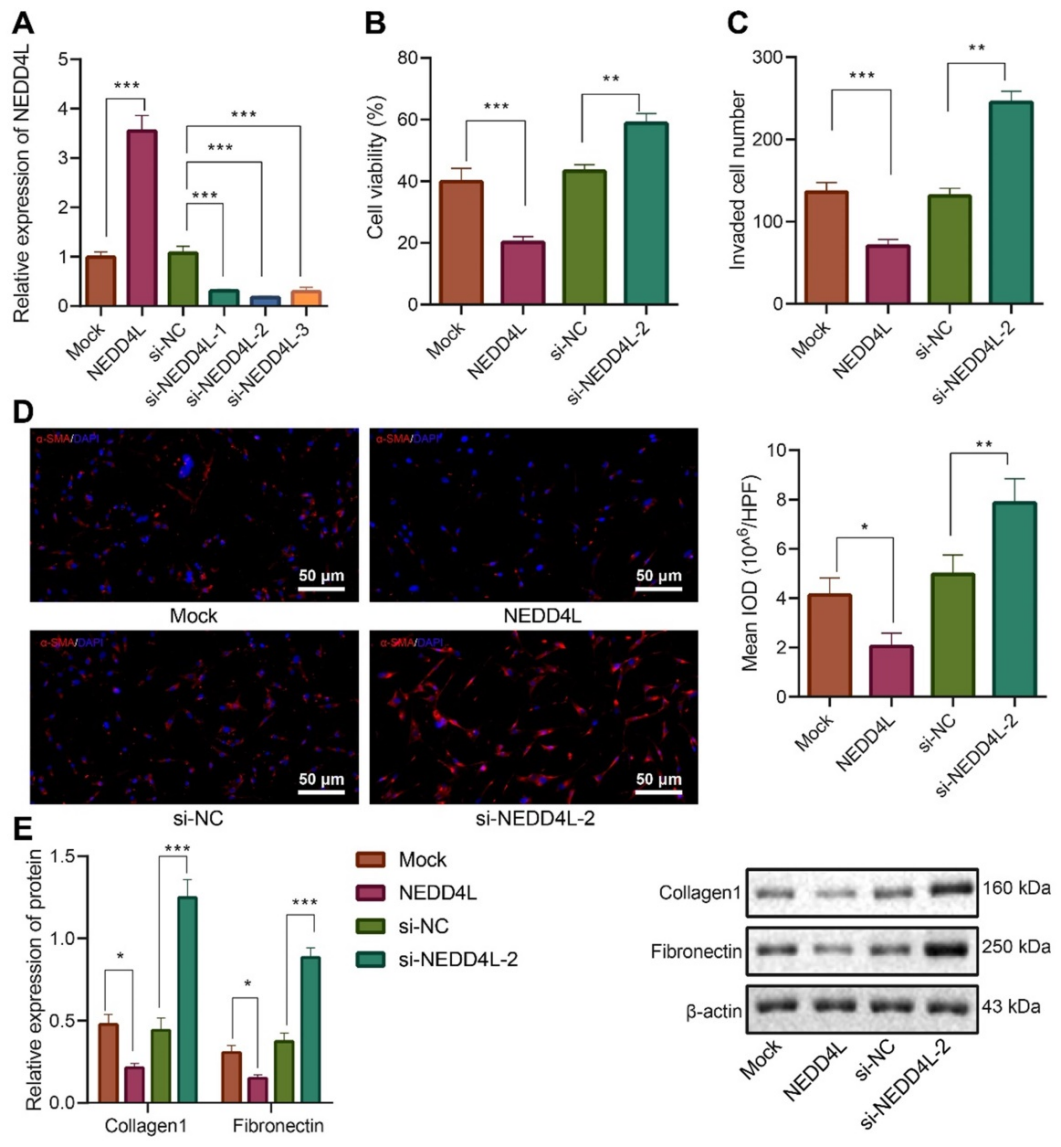
**Inhibitory effect of NEDD4L on the proliferative, invasive and differentiative abilities of LFs.** A: qRT-PCR to measure the expression of NEDD4L in LFs of each group; B: CCK-8 assay to detect the viability of LFs of each group; C: Transwell to evaluate the invasion of LFs of each group; D: Representative images and quantitative analysis of immunofluorescence to detect the level of α-SMA; E: Western blot to measure the levels of fibrosis-related proteins in LFs of each group. *** *p* < 0.001 versus the Mock group. Measurement data were summarized as mean ± standard deviation. Independent sample t test was applied for comparison between data of two groups; one-way ANOVA was adopted for comparison among data of multiple groups. The experiment was performed in triplicates and in the presence of TGF-β1.

After identifying the inhibitory effect of NEDD4L on IPF, we then explored the underlying molecular mechanisms, especially the potential regulatory effect of NEDD4L on LFs. Through manipulating NEDD4L expression in mouse LFs, we identified si-NEDD4L-2 with the best silencing efficiency and thereby selected it for subsequent experiments (Figure 2A). Further, the overexpression of NEDD4L resulted in suppressed viability (Figure 2B), invasion (Figure 2C), and myofibroblast marker α-SMA expression (Figure 2D); whereas silencing NEDD4L led to the opposite (Figure 2B, C, D). Then, through Western blot we found that the levels of fibrosis-related proteins (collagen 1, Fibronectin) were down-regulated and up-regulated, respectively, in the presence of NEDD4L overexpression and interference in LFs (Figure 2E). In summary, NEDD4L could alleviate IPF through attenuate the proliferative, invasive and differentiative abilities of LFs.

### NEDD4L inversely regulates the CTHRC1/HIF-1α axis to repress the proliferative, invasive and differentiative abilities of LFs

**Figure 3:**
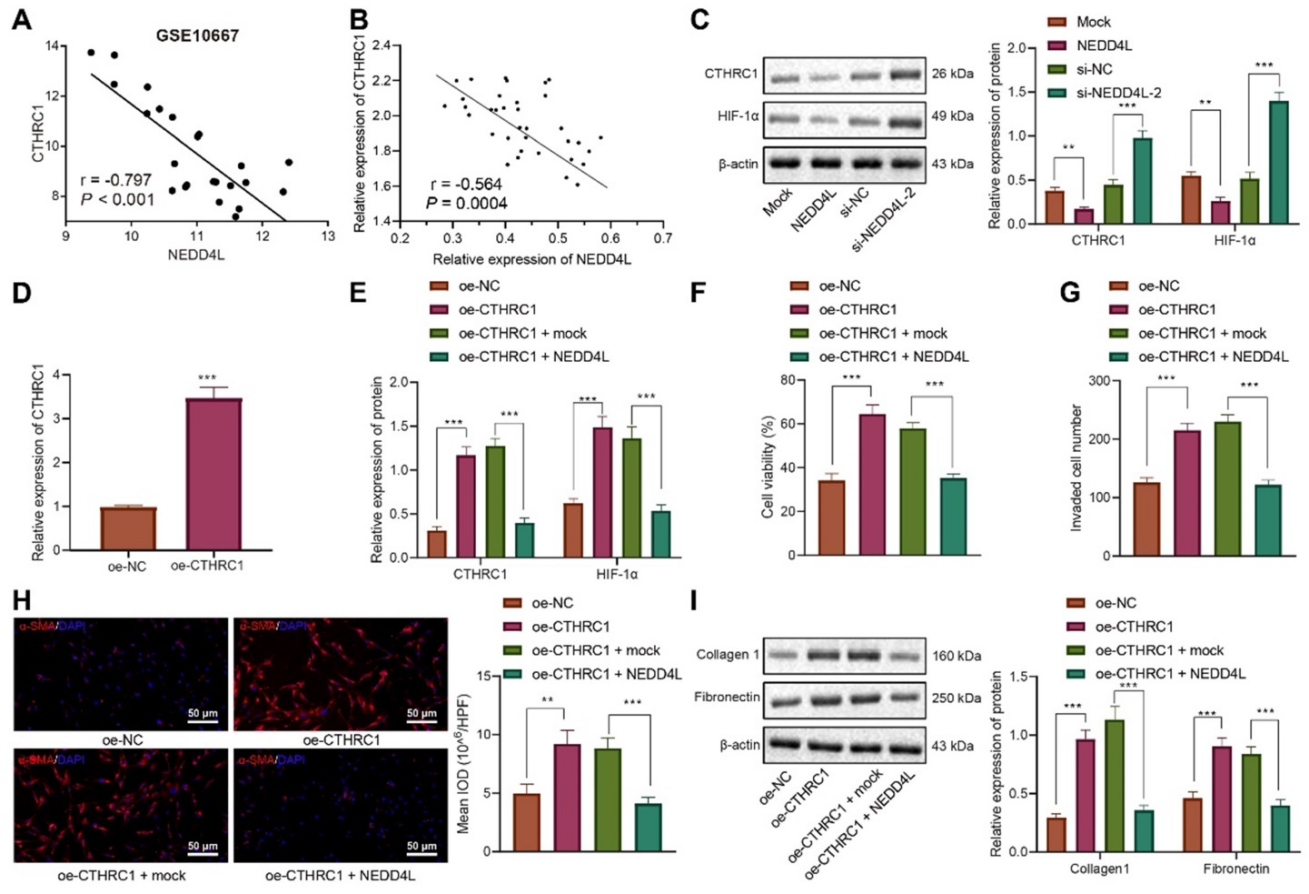
**NEDD4L regulates the CTHRC1/HIF-1α axis to repress the proliferative, invasive and differentiative abilities of LFs.** A: Negative correlation between NEDD4L and CTHRC1 expression in GSE10667 microarray; B: Pearson correlation analysis on NEDD4L and CTHRC1 expression in lung tissues of IPF patients (n = 35); C: Western blot to measure the protein levels of CTHRC1 and HIF-1α in LFs; D: qRT-PCR to determine the expression of CTHRC1 and HIF-1α in LFs of each group; F: CCK-8 assay to detect the viability of LFs; G: Transwell to detect the invasive ability of LFs; H: Immunofluorescence to measure the level of α-SMA in cells of each group; I: Western blot to determine the levels of fibrosis-related proteins in LFs of each group. * *p* <0.05, ** *p* < 0.01, *** *p* < 0.001 versus the Mock or oe-NC group. Measurement data were summarized as mean ± standard deviation. Independent sample t test was applied for comparison between data of two groups; one-way ANOVA was adopted for comparison among data of multiple groups. The experiment was performed in triplicates and in the presence of TGF-β1.

Further to explore the downstream mechanisms of NEDD4L modulating LFs, we found that NEDD4L was negatively correlated with CTHRC1 expression in lung tissue of IPF (Figure 3A). To validate our speculation that NEDD4L may modulate pulmonary fibrosis through the CTHRC1/HIF-1α signal axis, we performed Pearson correlation analysis on 35 lung tissue of IPF and revealed the negative correlation between the expression of NEDD4L and CTHRC1 (Figure 3B). Then, through Western blot, we substantiated that NEDD4L overexpression led to reduced levels of CTHRC1 and HIF-1α in LFs, whereas silencing NEDD4L led to increased levels of them (Figure 3C). Further, level of CTHRC1 in the LFs treated with plasmids overexpressing CTHRC1 was increased (Figure 3D), and CTHRC1 overexpression up-regulated the protein levels of CTHRC1 and HIF-1α, in contrast to the effect of NEDD4L overexpression (Figure 3E). The results of CCK-8, Transwell, immunofluorescence, and Western blot further indicated that overexpression of CTHRC1 could elevate the viability (Figure 3F), invasion (Figure 3G), and fibrosis-related proteins (Figure 3H, I) in LFs, while NEDD4L overexpression could attenuate the stimulatory effect of CTHRC1. Taken together, NEDD4L may modulate LFs through the CTHRC1/HIF-1α axis and thereby alleviate IPF.

### NEDD4L stimulates β-catenin ubiquitination and represses the activation of CTHRC1/HIF-1α axis

**Figure 4:**
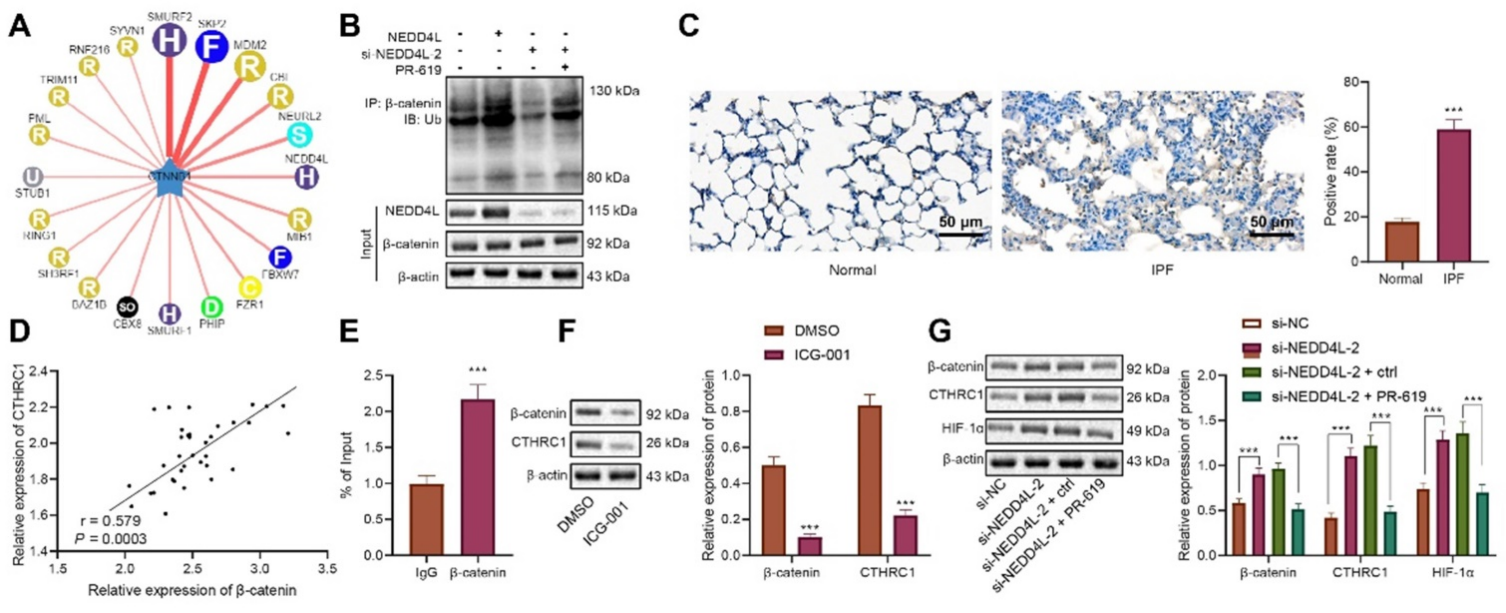
** NEDD4L stimulates β-catenin ubiquitination and thereby restricts the activation of the CTHRC1/HIF-1α axis.** A: E3 ubiquitin ligase of β-catenin (CTNNB1) predicted by UbiBrowser database; B: Immunoprecipitation to determine β-catenin ubiquitination in LFs; C: Immunohistochemistry to detect β-catenin expression in 35 IPF and 28 normal lung tissues (scale bar = 50 μm); D: Pearson correlation analysis on β-catenin and CTHRC1 expression in 35 cases of IPF lung tissues; E: ChIP to evaluate β-catenin enrichment in the promoter region of CTHRC1 gene; F: Western blot to determine levels of β-catenin and CTHRC1 proteins in LFs of each group; G: Western blot to determine the protein levels of β-catenin, CTHRC1 and HIF-1α in LFs of each group. * *p* <0.05, ** *p* < 0.01, *** *p* < 0.001 versus the Normal, IgG, DMSO, or si-NC group. Measurement data were summarized as mean ± standard deviation. Independent sample t test was applied for comparison between data of two groups; one-way ANOVA was adopted for comparison among data of multiple groups. The experiment was performed in triplicates.

Since the aforementioned experiments have indicated the inhibitory effect of NEDD4L on the CTHRC1/HIF-1α axis, we then explored the underlying mechanisms. Through bioinformatic analysis we found that NEDD4L may be the E3 ubiquitin ligase of β-catenin (CTNNB1) (Figure 4A). Manipulating the expression of NEDD4L in LFs, we revealed that NEDD4L overexpression led to augmented β-catenin ubiquitination, and vice versa (Figure 4B). These results suggested that NEDD4L may regulate the CTHRC1/HIF-1α signal axis by promoting the ubiquitination of β-catenin.

Further, with immunohistochemical staining we identified up-regulated level of β-catenin in lung tissue of IPF (Figure 4C). Person correlation analysis also indicated the positive correlation between β-catenin level and CTHRC1 expression in lung tissues of IPF patients (Figure 4D). The results of ChIP uncovered the enrichment of β-catenin in the promoter region of CTHRC1 gene (Figure 4E). Subsequently, we revealed that the levels of β-catenin and CTHRC1 were down-regulated in response to ICG-001, the β-catenin inhibitor (Figure 4F). These results indicated that β-catenin may activate the expression of CTHRC1 by binding to the CTHRC1 promoter region.

When silencing NEDD4L in LFs, the ubiquitination of β-catenin was repressed; while treatment of PR-619, a deubiquitinase inhibitor, led to increased β-catenin ubiquitination (Figure 4B). Further, through Western blot, we revealed that silencing NEDD4L resulted in up-regulated protein levels of β-catenin, CTHRC1, and HIF-1α in LFs, while PR-619 could reverse such up-regulation (Figure 4G). In summary, NEDD4L may stimulate β-catenin ubiquitination and thereby restrict the activation of the CTHRC1/HIF-1α axis.

### NEDD4L stimulates β-catenin ubiquitination, represses CTHRC1/HIF-1α axis, and alleviates IPF *in vivo*

**Figure 5:**
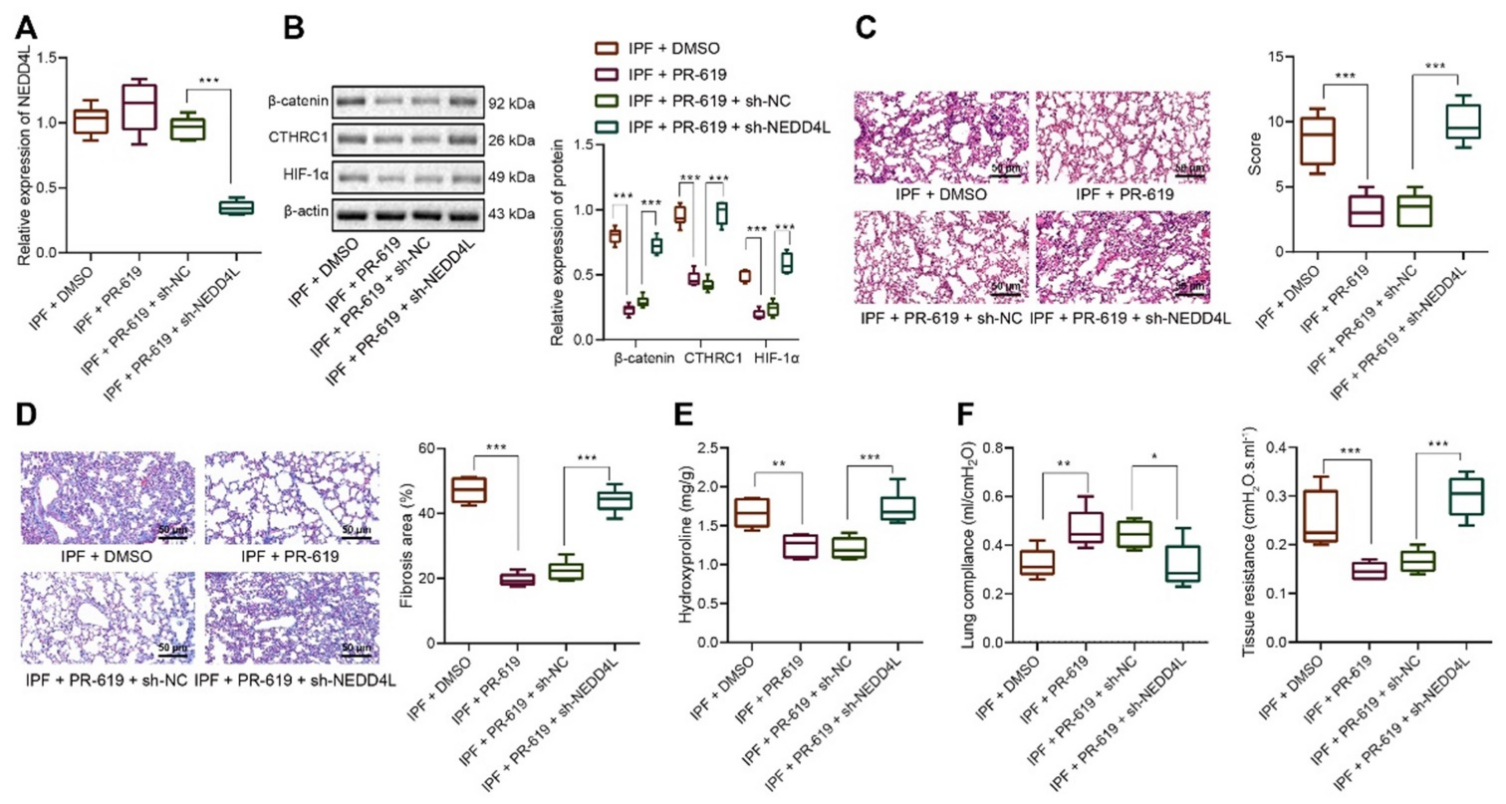
** NEDD4L stimulates β-catenin ubiquitination, represses CTHRC1/HIF-1α axis, and alleviates IPF *in vivo*.** A: qRT-PCR to measure the expression of NEDD4L in the lung tissues of mice treated with PR-619 and sh-NEDD4L; B: Western blot to measure the protein level of β-catenin, CTHRC1 and HIF-1α in the lung tissues of mice treated with PR-619 and sh-NEDD4L; C: Representative images and scores of the pathological changes in the lung tissues of mice treated with PR-619 and sh-NEDD4L (n = 6); D: Masson staining to examine lung tissue fibrosis in mice treated with PR-619 and sh-NEDD4L (n = 6); E: Detection of hydroxyproline content in the lung tissues of mice treated with PR-619 and sh-NEDD4L (n = 6); F: Resistance and compliance plethysmograph to detect lung functions of mice treated with PR-619 and sh-NEDD4L (n = 6). * *p* <0.05, ** *p* < 0.01, *** *p* < 0.001 versus the IPF + DMSO group. Measurement data were summarized as mean ± standard deviation. Independent sample t test was applied for comparison between data of two groups; one-way ANOVA was adopted for comparison among data of multiple groups. The experiment was performed in triplicates.

Following the *in vitro* substantiation of the regulatory mechanism of NEDD4L, we then moved to *in vivo* experiments. Our data unraveled that NEDD4L silencing led to down-regulated level of NEDD4L in lung tissues of PR-619-treated mice (Figure 5A). Results of Western blot further revealed down-regulated levels of β-catenin, CTHRC1 and HIF-1α in the lung tissues in response to PR-619, whereas the combination of PR-619 and sh-NEDD4L then led to up-regulated levels of them (Figure 5B). Moreover, we identified that PR-619 treatment could alleviate the lung tissue lesions of IPF mice (Figure 5C), repress pulmonary fibrosis (Figure 5D), reduce the content of hydroxyproline in tissues (Figure 5E), and resulted in up-regulated compliance and down-regulated resistance of the lung (Figure 5F), whereas sh-NEDD4L could reverse the aforementioned effect of PR-619. Altogether, these results indicated that NEDD4L may augment β-catenin ubiquitination, attenuate the activation of the CTHRC1/HIF-1α axis, and thus suppress IPF *in vivo*.

## Discussion

**Figure 6:**
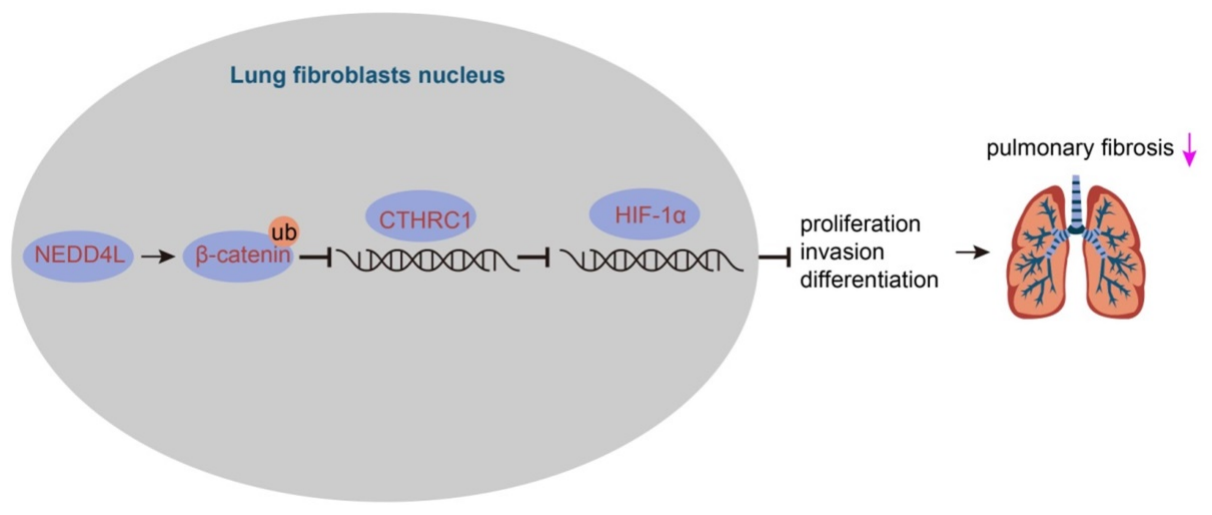
** The mechanism graph of the regulatory network and function of NEDD4L in pulmonary fibrosis.** NEDD4L stimulates β-catenin ubiquitination, represses the CTHRC1/HIF-1α axis, attenuates the biological potential of lung fibroblasts, and thus alleviates pulmonary fibrosis.

IPF represents a severe progressive interstitial lung disease with limited therapeutic options and poor prognosis 14. It is characterized by aberrant fibrotic remodeling of the distal lung, giving rise to lung dysfunction and ultimately death due to respiratory failure 15. Emerging data have suggested that myofibroblasts contributed to the accumulation of extracellular matrix in fibrotic diseases, and targeting the viability and differentiation of fibroblast has thereby been considered as a promising therapeutic strategy for IPF 5,6. In this study, we investigated the role of NEDD4L and substantiated that NEDD4L could augment β-catenin ubiquitination and attenuate the activation of CTHRC1/HIF-1α axis, thereby suppressing the biological activities of LFs in IPF (Figure 6).

Our initial finding of the down-regulated expression of NEDD4L in IPF-related expression microarrays indicated the involvement of NEDD4L in the pathogenesis of IPF. Then, our data revealed that NEDD4L overexpression repressed inflammatory cell infiltration and protect lung functions, thereby restricting the progression of IPF. This finding corroborates a previous report that the conditional deletion of NEDD4L in lung epithelial cells of IPF mice could trigger pulmonary fibrosis and exacerbate IPF, which also indicated that NEDD4L was mainly distributed in epithelial cells lining the distal airways of IPF patients 8. Further, fibroblasts have been recognized as the main cell type stimulating the accumulation of extracellular matrix in fibrotic diseases 16, which indicated the crucial role of LFs in IPF. On this basis, we investigated the influence of NEDD4L on LFs and our data substantiated that NEDD4L overexpression could attenuate the proliferative, invasive and differentiative abilities of LFs. Continuing to explore the downstream mechanisms of NEDD4L modulating LFs, we demonstrated that NEDD4L expression was negatively correlated with CTHRC1 expression in lung tissues of IPF. CTHRC1, a secreted protein, has been found to be implicated in a series of physiological and pathological processes, including inflammatory arthritis, vascular remodeling, bone formation, and cancer development. Of note, in agreement with our data, a recent case has suggested that the level of CTHRC1 was elevated in lung tissues of IPF, and it also indicated that CTHRC1 could promote pulmonary fibrosis through inducing LFs activity 9.

Then, through a series of gain- and loss- of function experiments we found that NEDD4L inversely regulated the CTHRC1/HIF-1α axis to repress the proliferative, invasive, and differentiative abilities of LFs. In relation to this, it has been pointed out that CTHRC1 could up-regulate the expression of HIF-1α in gastric cancer 10. HIF-1α, a key mediator in cell metabolism and inflammation under hypoxic conditions, has recently been highlighted for functioning as a trigger of pulmonary fibrosis and also other types of fibrosis 11,17-19. Moreover, our data revealed that NEDD4L repressed the activation of CTHRC1/HIF-1α axis *via* stimulating β-catenin ubiquitination. This corroborates previous studies which indicated that β-catenin could bind to the promoter region of CTHRC1 20,21. Also, accumulating data have established that the Wnt/β-catenin signaling pathway was activated in pulmonary fibrosis and was correlated with the viability and differentiation of LFs 12,22,23. Afterwards, our *in vivo* experiments further validated that NEDD4L stimulated β-catenin ubiquitination, repressed the CTHRC1/HIF-1α axis, and thus alleviated IPF.

Taken together, the evidence provided in this study has led us to a conclusion that NEDD4L could suppress the formation and progression of IPF *via* augmenting β-catenin ubiquitination and inhibiting the CTHRC1/HIF-1α axis. By inducing the ubiquitination of β-catenin, NEDD4L could repress the activation of the Wnt/β-catenin signaling pathway, and restrict the CTHRC1/HIF-1α axis due to the CTHRC1 expression-promoting effect of β-catenin; then, CTHRC1 and HIF-1α suppression could attenuate the viability and differentiation of LFs, thereby impeding the formation and progression of IPF. In summary, this study elucidated the regulatory network of NEDD4L in IPF, deepened our understanding of molecular mechanisms involved in the progression of IPF, and, more importantly, provided promising therapeutic targets for IPF treatment.

## Supplementary Material

Supplementary table.

## Abbreviations

Interstitial pulmonary fibrosis): IPF
lung fibroblasts): LFs
Neural precursor cell expressed developmentally down-regulated 4-like protein): NEDD4L
collagen triple helix repeat containing protein 1): CTHRC1
