# Supplementary material for: NEDD4L-induced β-catenin ubiquitination suppresses the formation and progression of interstitial pulmonary fibrosis via inhibiting the CTHRC1/HIF-1α axis
Source: Int J Biol Sci. 2021 Jul 25;17(13):3320–30. doi: 10.7150/ijbs.57247 (PMC8416742; doi:10.7150/ijbs.57247)
Supplement: Supplementary file 1 — Supplementary table. [file ijbsv17p3320s1.pdf]

Supplementary Table 1 Primer sequences for qRT-PCR

Genes	Primer sequences
NEDD4L (human)	Forward: 5’-AGAAACTGCCCAGAGCTCAC-3’
	Reverse: 5’-TCGCCTCTGCAAAAGTCTGT-3’
NEDD4L (mouse)	Forward: 5’-CAACTTGGACTCGGCCAATC-3’
	Reverse:5’-GTTACTGTTGGCGAGCTGAG-3’
CTHRC1 (human)	Forward: 5’-ATAATGGAATGTGCTTACAAGG-3’
	Reverse: 5’-TTCCCAAGATCTATGCCATAAT-3’
CTHRC1 (mouse)	Forward: 5’-CTGCTACAGTTGTCCGCACC-3’
	Reverse: 5’-GGTCCTTGTAGACACATTCCATT-3’
GAPDH (human)	Forward: 5’-CATCCATGACAACTTTGGTATCGT-3’
	Reverse: 5’-CAGTCTTCTGGGTGGCAGTGA-3’
GAPDH (mouse)	Forward: 5’-TGTCCGTCGTGGATCTGAC-3’
	Reverse: 5’-CCTGCTTCACCACCTTCTTG-3’

Note: NEDD4L, neural precursor cell expressed developmentally down-regulated 4-like protein; CTHRC1, collagen triple helix repeat containing protein 1; GAPDH, glyceraldehyde-3-phosphate dehydrogenase.
